# Development and psychometric testing of a Chinese version of the postnatal care experience scale for postpartum women

**DOI:** 10.1186/s12884-023-06187-z

**Published:** 2023-12-16

**Authors:** Liping Sun, Xiaojiao Wang, Hua Gao, Zhaorun Li, Meiyi Chen, Xu Qian, Chunyi Gu

**Affiliations:** 1 Department of Nursing, Obstetrics and Gynecology Hospital of Fudan University, Shanghai, China; 2 Department of Maternal, Child and Adolescent Health, School of Public Health, Fudan University, Shanghai, China; 3 Global Health Institute, Fudan University, Shanghai, China

**Keywords:** Experience, Measurement, Postnatal care, Psychometrics, Scale

## Abstract

**Background:**

Postnatal period is a critical transitional phase in the lives of mothers and newborn babies. In recent years the importance on promoting a positive experience of care following childbirth is increasingly emphasized. Yet published evidence of the methodological and psychometric quality of instruments to evaluate women’s experience of comprehensive postnatal care is still lacking.

**Objective:**

This study aimed to develop and validate a unique scale (the Chinese version of the Postnatal Care Experience Scale, PCES) to measure women’s overall experience of care during postnatal periods.

**Methods:**

The PCES instrument was developed and validated over three phases, including item development, scale development, and scale evaluation. The item pool of the PCES was generated through existing literature and in-depth semi-structured interviews, followed by assessment of content validity and rating of importance and feasibility of items through two-round Delphi surveys. Psychometric properties were examined in a convenience sample of 736 postpartum women. Both exploratory factor analysis (EFA) and confirmatory factor analysis (CFA) were conducted to assess the construct validity of the developed PCES. The relationship between the total PCES score and the global item construct was estimated using Pearson product-moment coefficient. Reliability was assessed using Cronbach’s alpha and Spearman Brown coefficients.

**Results:**

The content validity index of the Chinese version PCES was 0.867. Following item reduction analysis, this instrument consisted of 30 five-point Likert items. The Kaiser-Meyer-Olkin statistic was 0.964 and the chi-square value of the Bartlett spherical test was 11665.399 (*P* < .001). The scale explained 75.797% of the total variance and consisted of three subscales, including self-management, social support, and facility- and community-based care. The Pearson correlation coefficient between the total PCES score and the global item construct was 0.909. The CFA showed that the 3-factor model had suitable fitness for the data. Cronbach’s alpha value and Spearman-Brown Split-half reliability for the total scale were 0.979 and 0.941, respectively.

**Conclusions:**

The newly developed 30-item PCES is a psychometrically reliable and valid instrument that assesses women’s overall experience of postnatal care. Future research should aim to use the PCES in various populations to obtain further evidence for its validity and reliability.

## Background

The terms “postnatal period” and “postpartum period” are often used interchangeably. The postnatal period is defined by the World Health Organization as one that begins immediately after the birth of the baby and extends up to six weeks (42 days) after birth [1]. This period is a critical transitional phase in the lives of mothers and newborn babies [2, 3]. According to statistics, one third of pregnancy-related deaths occur between 1 week and 1 year after birth; and one fifth occur between 7 and 42 days postpartum [4]. In the past few decades, China has achieved a remarkable decrease in both the maternal mortality ratio (MMR) (from 88.8 per 100,000 live births in 1990 to 18.3 per 100,000 live births in 2018) and the neonatal mortality ratio (NMR) (from 33.1 deaths per 1000 live births in 1991 to 3.9 deaths per 1000 live births in 2018) [5]. However, given the large population in China, it still lags behind some developed countries such as Australia, Germany and Japan [6, 7]. Alongside severe maternal morbidities, the postnatal period could present considerable challenges for women, including recovering from childbirth, learning to care for herself and her baby, lack of sleep, fatigue, pain, breastfeeding difficulties, stress, mental health disorders, lack of sexual desire, urinary incontinence and other chronic health conditions [3, 4, 8–10].

Therefore, the postnatal period poses substantial health risks for both mother and newborn infant. Care during this time is critical not only for survival but also to the long-term health and well-being of mothers and infants. Yet the postnatal period is the most neglected period for the provision of quality care and receives less attention from health care providers than pregnancy and childbirth both in China and globally [2, 11]. For instance, in the United States, many women receive little formal or informal maternal support for both their recovery and infant care, and are expected to quickly mobilize during the postnatal period [3]. Conversely, postpartum women in China participate in a traditional ancient practice called “zuoyuezi” or “doing the month” for postnatal care. They are required to stay at home for a month immediately after childbirth, which helped acknowledge the woman’s societal and familial contribution to childbearing and the provision of consistent family support [12].

Nevertheless, one cohort study conducted in Shanghai showed that not all the activities of “doing the month” provided protection for women, emphasizing the importance of allowing flexibility to fit and adjust the ritual into the modern life [13]. In addition to family support, the community, outreach and facility-based care are all the essential postnatal health service delivery ways. The Chinese national policy and guidelines have emphasized that at least one postnatal home visit for mothers and infants within one week after delivery, followed by a facility healthcare visit for them within 42 days after delivery were needed [11]. However, studies have shown that the coverage and quality of postnatal care were low in China [11, 14]. Li et al. found that only 8% of women received a timely postnatal home visit (within one week after birth) and 24% received postnatal care within 42 days after birth [11]. Understaffing, lack of funding and inadequate training on postnatal care are the main barriers that affect provision of postnatal care [11, 14, 15]. Consequently, postnatal care is the area of maternity care that is most in need of improvement.

Experience of care is an integral part of quality healthcare and perceived quality of care is an important determinant of service utilization [16, 17]. The importance on promoting a good “experience of care” following childbirth is increasingly emphasized in recent documents, such as the “WHO recommendations on maternal and newborn care for a positive postnatal experience” [18]. Women play a central role in assessing and defining quality of care based on their previous experiences of maternal health services [17, 19–21]. Evidence shows that satisfied women are more likely to comply, return for care, and have better outcomes [22, 23], while negative experiences of care may act as a deterrent to current or future utilization of health services [24]. Hence, the measurement of postnatal care experience in health facilities should be carried out regularly as a basic indicator to evaluate and improve the quality of postnatal care for women and newborns [25, 26].

In China, postnatal care of the mother and newborn begins in the hospital following childbirth and continues at home visits and outpatient clinics with different care providers. However, this early health care following childbirth has not been evaluated as a unified episode in the continuum of care and has rarely been systematically evaluated [26, 27]. Published evidence of the methodological and psychometric quality of self-report instruments to evaluate women’s experience of maternity care especially postnatal care is lacking [28]. Although several scales measuring evaluation of postnatal care services have been developed and validated in the international literature [23, 26, 29], these instruments are specifically for hospital postnatal care and are not suited for evaluating comprehensive health care following childbirth (including inpatient hospital care, outpatient care, community health services and family care) across different health providers. In the absence of a valid and reliable instrument to assess the maternal experience of postnatal care in China, we sought to develop a new scale that could measure this concept. Identifying women’s overall experience of care during postnatal periods and raising awareness of continuum of care following childbirth are important for the improvement of quality of postnatal care in both hospital and community settings. Thus, this study presents the assessment of validity and reliability of a Chinese version of the Postnatal Care Experience Scale (PCES), enabling the health care providers to use this tool to evaluate and improve the quality of comprehensive postnatal care to all women and their newborns.

## Methods

**Fig. 1:**
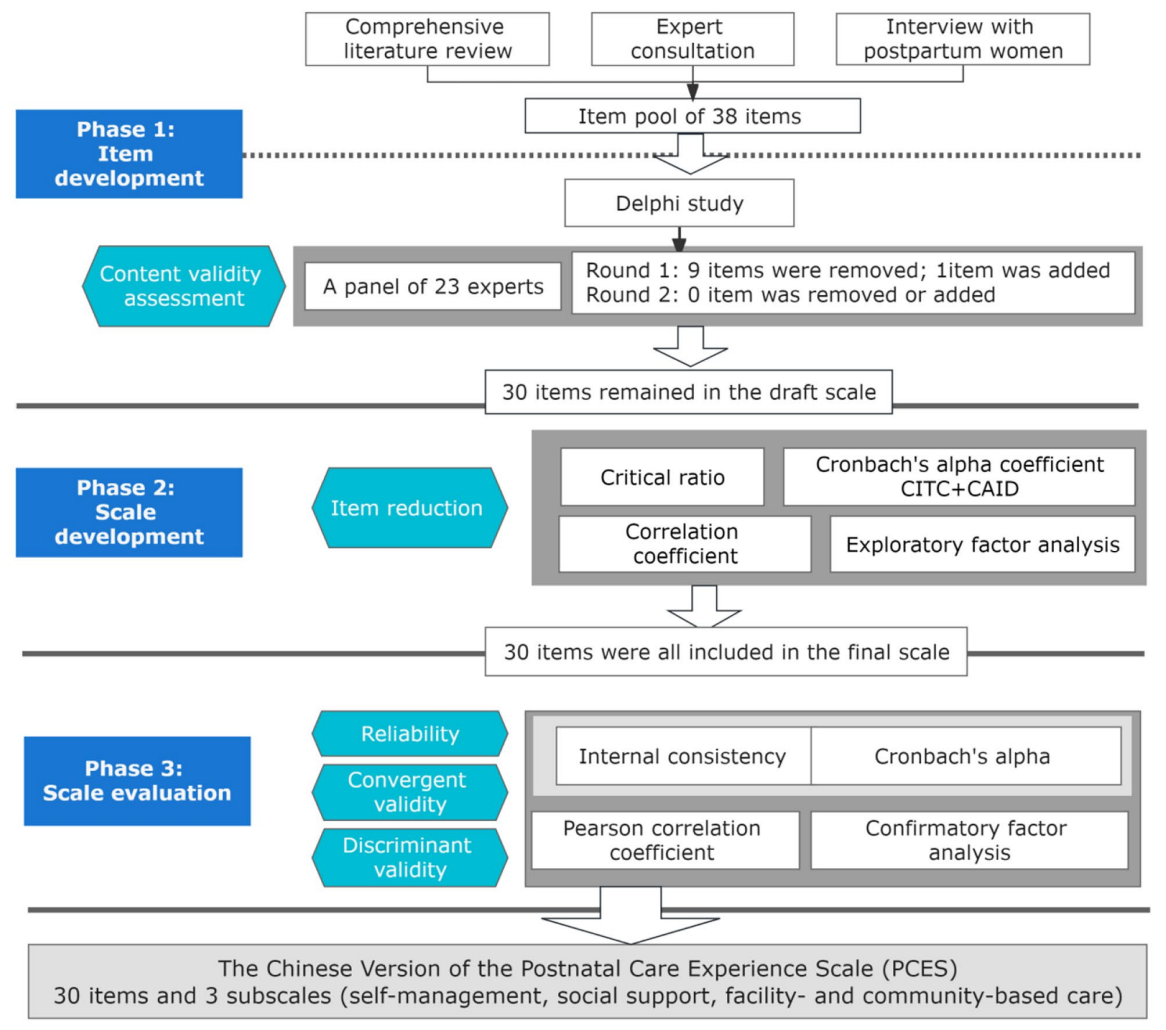
Process of development of the Chinese version of the Postnatal Care Experience Scale (PCES)

The aim of this study was to develop and validate the Chinese version of Postnatal Care Experience Scale (PCES) to measure maternal experience of care during postnatal periods. Specific aims were comprised of three phases. “Phase 1 item development” was to generate items for the PCES and to conduct content validity assessment; “Phase 2 scale development” was to perform exploratory factor analysis of the PCES to determine final items; and “Phase 3 scale evaluation” was to conduct psychometric testing of the final version of the PCES (Fig. 1). Ethical approval was received from the Hospital Ethics Committee of Obstetrics and Gynecology Hospital of Fudan University (OGHEC2021107).

***Phase 1: Item Development***.

Our scale development process was guided by Boateng et al.’s primer of best practices for developing and validating scales for health, social, and behavioral research [30]. The initial item pool of the PCES was developed based on the relevant literature and documents on postnatal care [31–34]. A draft form with 42 items was created. Five experts in the field of maternity care and six postpartum women were invited to evaluate the draft scale through face-to-face, semi-structured, in-depth individual interviews. They were asked to assess the items’ suitability for the required topics, and the necessity, readability, and clarity of each item. Qualitative data obtained from these interviews were transcribed. Transcripts were repeatedly read by the researchers. Each statement extracted from transcripts was assessed for its obvious and hidden meanings. Quotations reflecting the views and perceptions of experts and postpartum women about postnatal care services were refined. The research team held two consensus meetings to examine the quotations and expressions and then decided which items to include in the item pool. Based on feedback from the interviewees, a new item was added; 2 items were modified; and 5 items were removed from the item pool of 42 items, making the final item pool 38 items. A 5-point Likert scale ranging from 1 = strongly disagree to 5 = strongly agree was used in the response options.

To further optimize the initial PCES, two rounds of Delphi surveys were conducted between 7 September and 31 October 2020. A purposive non-probability sampling was used to ensure that the appropriate experts were invited to participate. A group of experts was established based on the following criteria: (1) at least 10 years of working experience as a professional in maternal healthcare, obstetrics, nursing and midwifery; (2) with senior professional titles; and (3) being familiar with postnatal care practice. The Delphi questionnaire consisted of four parts: (1) experts’ demographic characteristics, (2) description of the PCES’s purpose and instructions for assessing content validity, (3) the initial PCES scale and (4) experts’ self rated authority and their familiarity with items. During each round, experts were provided with a link to the survey and they were required to rate the importance and feasibility of each item on a 5-Likert scale. All items that were rated less than 4.0 by any expert were deleted. The first Delphi round was completed within 3 weeks. Items were either accepted, rejected, modified or merged based on authors’ discussion considering experts’ comments [35]. During the second round, the revised scale was sent back to the experts for comment and scoring. Consensus among the expert panel was achieved after two-round Delphi process. The coefficient of variation (CV) and Kendall harmony coefficient (W) were used to reflect the coordination level of expert opinions. It was predetermined that items with CV ≤ 0.25 were included in the scale. We calculated the scale-content validity index with universal agreement (S-CVI/UA) to assess the content validity. The scale was considered to have good content validity with a S-CVI/UA of greater than or equal to 0.80 [36, 37].

Face validity of the PCES was assessed through qualitative interviews with 12 postpartum women who were the target group of the PCES. Participants were invited to read the scale carefully and express their understanding of the total scale and each item of the PCES. The items of the scale would be revised according to women’s suggestions and opinions.

***Phase 2: Scale development***.

In scale development, item reduction analysis is conducted to ensure that only parsimonious, functional, and internally consistent items are finally included [30]. After the two-round Delphi surveys, the 38-item draft scale were modified and merged into a 30-item scale. The PCES had 30 self-report items with a 5-point Likert scale response format that ranged from strongly disagree (1) to strongly agree (5). Participants were asked to indicate their level of agreement with the items. Higher scores reflected a more positive experience towards postnatal care. The PCES total score was calculated with the summation of all responses from the 30 items, with a minimum score of 30 and maximum score of 150. We conducted exploratory factor analysis (EFA) to further ascertain the number of items in the PCES by eliminating any that were redundant or not congruent with the overall construct being measured.

### Setting and sample

We recruited a convenience sample of at least 300 women to participate in the item reduction step. The sample size of the field investigation was determined based on the principle of the sample size being at least 5 to 10 times the number of measured items [38, 39]. DeVellis suggests that a sample size of 200 is adequate in most cases of factor analysis [40], while Comrey and Lee state that a sample size of 300 is good [41].

A sample of 343 postpartum women were recruited from the Obstetrics and Gynecology Hospital of Fudan University in Shanghai, China between 1 November, 2020 and 31 January 2021. This hospital is a specialised tertiary teaching hospital in China, which consists of two campuses (Huangpu campus and Yangpu campus) and the total number of births annually is approximately 10,000. The woman who has given birth in the hospital will be informed to attend the postnatal outpatient clinic at 42 days postpartum. Besides, one healthcare provider at the community health center performs two home visits for the woman within 42 days following childbirth. In our study, women were eligible to participate if they met the following the criteria: (1) had given birth to a singleton live infant; (2) were 18–49 years of age; (3) had completed the postnatal outpatient visit at 42 days postpartum; (4) could read and write in Chinese. Women with an intellectual disability and a known psychiatric disorder and women who declined to participate in the study were excluded.

### Data collection

The nursing staff of the postnatal clinic were asked to identify participants who met the inclusion criteria. Women were then approached by the research assistants (ZRL, MYC, LPS) who provided a verbal explanation and informed written consent information about the study. Signed, informed written consent was obtained from those who were willing to participate. They were required to complete a brief sociodemographic form and the PCES.

### Data analysis

Four item screening methods were used to screen the scale items of the PCES. Firstly, the *Critical ratio (CR) method* was conducted to investigate the item sensitivity of the PCES. And 27% of the total score of the scale was used as the cut-off value to divide the PCES into high-score and low-score groups [42]. Independent sample t test was performed to compare whether the difference between high-score and low-score groups was statistically significant. The scale items would be included if the critical value was greater than 3.0 and the score difference between the two groups was statistically significant [43]. Secondly, the *correlation coefficient* between the score of each item and the total score of the PCES was measured. Items with correlation coefficient less than 0.4 were then removed [44]. Thirdly, we used *Cronbach’s alpha coefficient* to examine the internal consistency of the scale items. The corrected item-total correlation (CITC) was estimated to examine the correlation between the item and the sum score of the rest of the items excluding itself. Items with very low CITCs (< 0.30) are less desirable and could be a cue for potential deletion from the tentative scale [30]. Cronbach’s alpha if item was deleted (CAID) was also measured. The scale item was removed if CAID increased after deleting this item. Finally, *exploratory factor analysis (EFA)* was used to explore the underlying dimensions of the construct of interest. The item would be included based on the screening results of the five statistics (critical ratio, correlation coefficient between item and total score, CITC, CAID, and factor loading). Items that failed to meet the standard statistics (more than twice) were then deleted [42].

***Phase 3: Scale evaluation***.

Phase 3 involved administering the 30-item PCES to women to establish its construct validity and internal consistency reliability. Construct validity was evaluated using exploratory factor analysis (EFA) and confirmatory factor analysis (CFA). Principal component analysis was performed for EFA of all items and the Varimax rotation was carried out to evaluate the dimensionality of the PCES. The minimum acceptable factor load was determined to be 0.30 [30]. Prior to factor analysis, the Kaiser-Meyer-Olkin (KMO) test and the Bartlett spherical test were performed to assess the factorability of the dataset. When the KMO value exceeded 0.5, the sample size was adequate for factor analysis [43].

A separate sample of 393 postpartum women completed the PCES between 1 February, 2021 and 31 May, 2021. The CFA was performed to measure the model fitness. The model fit was checked using several fit indices including Chi-square and degrees of freedom ratio (χ^2^ /df ), root mean square error of approximation (RMSEA), goodness of fit index (GFI), comparative fit index (CFI), tucker lewis index (TLI), and incremental fit index (IFI). Values of 3.0 or below for χ^2^/df, values of 0.90 or more for GFI, CFI, TLI, and IFI, values of 0.08 or below for RMSEA, indicate acceptable model fit [45]. We assessed the construct reliability, convergent validity, and discriminant validity of the constructs. Construct reliability greater than 0.7 is an acceptable reliability coefficient [46]. The average variance extracted (AVE) scores were calculated as an estimate of convergent validity with values ≥ 0.50 considered to be acceptable [46]. Discriminant validity was confirmed when the square root of the AVE scores were higher than the correlations values across constructs.

Additionally, to examine the convergent validity of the scale, an extra global item “As a whole, I felt that my postnatal care experience was very good” was added at the end of the instrument. The item is scored with the following options: (1 = strongly disagree, 2 = disagree, 3 = neutral, 4 = agree, 5 = strongly agree). The relationship between the PCES scores and the global item construct was estimated using Pearson product-moment coefficient. The higher correlation coefficients suggest support for convergent validity [30].

Cronbach’s alpha was calculated to assess the reliability of the PCES, determining the internal consistency of the scale items [47, 48]. An alpha coefficient of 0.70 has often been regarded as an acceptable threshold for reliability, while 0.80 and 0.95 is preferred for the psychometric quality of scales [48, 49]. In addition, item-to-total correlation coefficients for the instrument were examined, as well as whether the Cronbach’s alpha increased if any of the items were deleted. The IBM SPSS Statistics v26.0 was used for all statistical analyses, except CFA. The CFA was performed using the AMOS software v28.0.

## Results

A total of 23 experts completed two rounds of Delphi process and achieved consensus. The effective response rate was 100% (23/23) in both rounds, indicating a high positive coefficient of the experts. The average age of experts was (43.17 ± 6.58) years and their experience in the relevant fields was (21.43 ± 7.26) years. Among them, 100% (23/23) held senior-level professional titles and 56.5% (13/23) obtained the educational level of master or doctorate degree. Experts’ coefficient of determination (Ca) was 0.870, and the degree of familiarity (Cs) was 0.920. The authority coefficient of experts (Cr= (Ca + Cs)/2) was 0.900, indicating the high reliability of this Delphi process.

In Round 1, nine items were removed, one item was added, and six items were modified in wording, leaving 30 items remained in the scale for Round 2. The Kendall harmony coefficient (W) in the first round of consultations was 0.281 (*P* < .001) in importance and 0.326 (*P* < .001) in feasibility. After two rounds of the Delphi survey, the final consensus scale consisted of 30 items, with the mean scores of the 30 items ranging from 4.39 to 5.00 points in importance and 4.52 to 4.91 points in feasibility, respectively. The Kendall harmony coefficient (W) in the second round of consultations was 0.299 (*P* < .001) in importance and 0.428 (*P* < .001) in feasibility. The CV of each item was between 0.00 and 0.15 in importance and between 0.06 and 0.17 in feasibility. The S-CVI of the PCES exceeded the a priori minimum of 0.80 with a S-CVI/UA of 0.867. For face validity, the 12 interviewed women reported that it was easy for them to read and understand the scale items. Thus there was no necessity to conduct any changes at this stage.

Overall, 736 pregnant women participated in the study. The age of respondents ranged from 18 to 44 years, with an average age of 30.85 years (SD = 3.74). The majority of women were Han ethnic (98.91%), had bachelor degree (55.84%) and Shanghai household registration (63.32%). The participants’ demographics and clinical characteristics are summarized in Table 1.

**Table 1:** Demographic and clinical characteristics of the study sample (n = 736)

Variables		Total sample (n = 736) Number (%)	EFA sample (n = 343) Number (%)	CFA sample (n = 393) Number (%)
**Age (years)**, [mean(SD)]		30.85 (3.74)	31.25 (4.10)	30.44 (3.12)
**Educational level**				
	Less than junior middle school	31 (4.21)	17 (4.96)	14 (3.56)
	High or technical secondary school	49 (6.66)	24 (7.00)	25 (6.36)
	Junior college	141 (19.16)	66 (19.24)	75 (19.08)
	Bachelor degree	411 (55.84)	189 (55.10)	222 (56.49)
	Master degree or above	104 (14.13)	47 (13.70)	57 (14.51)
**Nationality**				
	Han ethnic	728 (98.91)	341 (99.42)	387 (98.47)
	Minority	8 (1.09)	2(0.58)	6 (1.53)
**Registered residence**				
	Shanghai	466 (63.32)	211 (61.52)	255 (64.89)
	Other provinces and cities	270 (36.68)	132 (38.48)	138 (35.11)
**Mode of delivery**				
	Vaginal birth	483 (65.63)	226 (65.89)	257 (65.39)
	Cesarean section	253 (34.37)	117 (34.11)	136 (34.61)
**Newborn gender**				
	Male	380 (51.63)	174 (50.73)	206 (52.42)
	Female	356 (48.37)	169 (49.27)	187 (47.58)
**Neonatal birth weight (g)**, [mean(SD)]		3215.15 (511.75)	3293.47 (474.27)	3140.57 (522.99)

Complete responses were provided to all PCES items. There were no missing data. Table 2 shows that the critical ratios of all 30 items were > 3.0. Item-to-total correlation coefficients were positive (all *P* < .001) (Table 2). The CITC of each scale item was greater than 0.45. The CAID did not increase if any of the items were deleted, with the exception of item 1 “I feel that I can cope well with the postpartum recovery process” and item 2 “I feel that I can cope well with taking care of my baby”, showing a slight increase (Table 2).

**Fig. 2:**
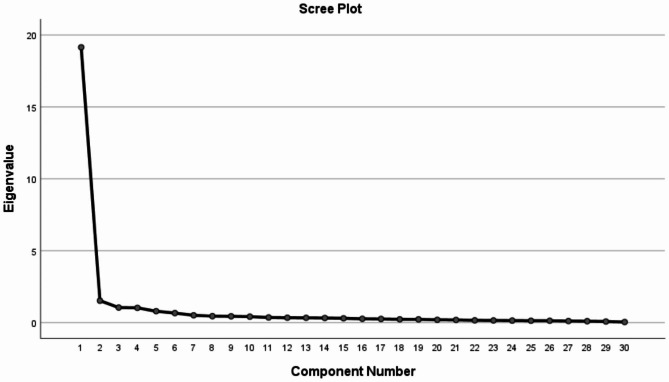
Scree plot for determining factors of the PCES instrument

The exploratory factor analysis was conducted with data from 343 subjects. The KMO statistic was found to be 0.964 (Table 3). The chi-square value of the Bartlett spherical test was 11665.399 (*P* < .001) (Table 3), indicating that it was suitable for conducting factor analysis. Principal component analysis using the Varimax rotation was used to extract the factor with eigenvalue > 1. The items were distributed according to the subfactors and the factor loadings of all items exceeded 0.30 (Table 4). Based on the comprehensive analysis of the five statistics (critical ratio, correlation coefficient between item and total score, CITC, CAID, and factor loading), all the 30 items of the PCES were included. Factor analysis of the 30-item scale yielded four factors which explained 75.797% of the total variance of the PCES, including factor 1 (items 1, 2), factor 2 (items 18, 19), factor 3 (items 3, 6, 7, 8, 9), and factor 4 (items 4, 5, 10–17, 20–30). The factor loads ranged between 0.530 and 0.853 for all items (Table 4). Figure 2 shows the scree plot of the PCES instrument.

The researchers examined the extracted four factors and grouped them in the most meaningful way based on clinical knowledge and experience. To ensure the measurement stability, scale items should be no less than three under each subscale [43]. Thus, the two items (18, 19) within factor 2 were merged into factor 4 due to their similarities in item description and measurement purpose. Likewise, considering the practical significance of measurement, item 3 and 9 within factor 3 were incorporated into factor 1 and factor 4, respectively; whereas item 4 and 5 within factor 4 were reclassified to factor 3. Therefore, the extracted four factors were finally grouped into three subscales, which was named as subscale 1 “Self-management” (items 1–3); subscale 2 “Social support” (items 4–8); and subscale 3 “Facility- and community-based care” (items 9–30).

**Fig. 3:**
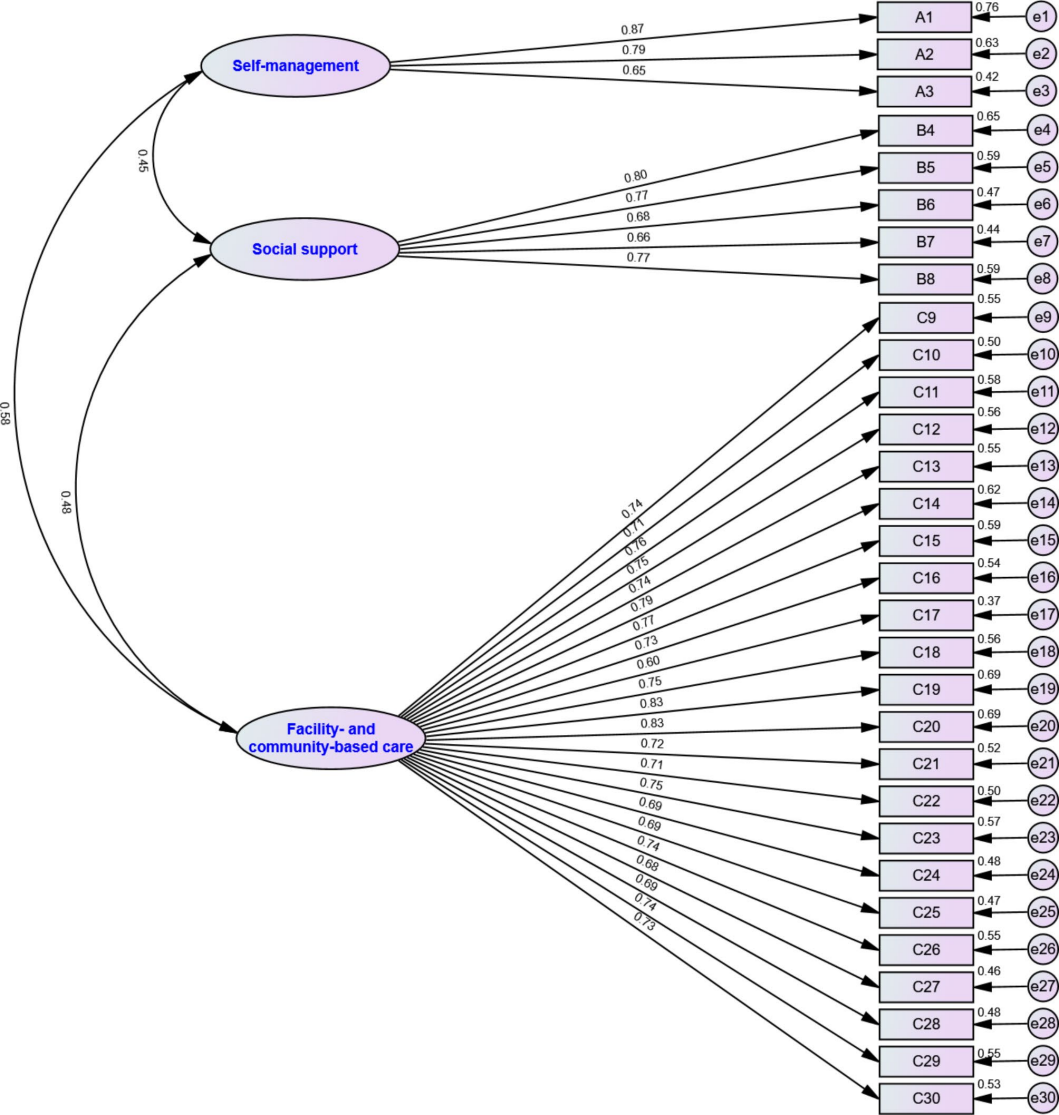
CFA standardised item loadings and factor correlations for the PCES instrument (n = 393)

The findings of the CFA of the general model with 30 items in three subscales indicated that the three-factor model had an acceptable fit, with χ^2^/df = 1.905 (χ^2^ = 765.7, df = 402), RMR = 0.028, RMSEA = 0.048, GFI = 0.885, TLI = 0.946, CFI = 0.950, IFI = 0.950. The construct reliability values of all three constructs were 0.818, 0.857, and 0.963, indicating good reliability. The AVE values for factor 1, factor 2, and factor 3 were 0.604, 0.546, and 0.540, respectively. The correlations values across constructs were all lower than the square root of the AVE scores, with $\sqrt{AVE}_{(F1)}=0.777$, $\sqrt{AVE}_{(F2)}=0.739$, $\sqrt{AVE}_{(F3)}=0.735$. Figure 3 shows the CFA standardised item loadings and factor correlations for the PCES instrument.

The PCES’s reliability was estimated using Cronbach’s alpha and Spearman Brown Split-half tests. Table 5 shows excellent internal consistency reliability for the overall scale (Cronbach’s alpha = 0.979, half-split reliability = 0.941). The Cronbach’s alpha and Spearman-Brown Split-half reliability were 0.709 and 0.624 for the self-management subscale; 0.881 and 0.860 for the social support subscale; and 0.980 and 0.952 for the facility- and community-based care subscale, respectively (Table 5). The Pearson correlation coefficient between the total PCES score and the global item construct was 0.909 (Table 5).

**Table 2:** The scale item screening results of critical ratio, correlation coefficient, CITC, and CAID

Subscales	Item	Critical ratio		Correlation coefficient		CITC	CAID
		CR	*P v*alue	R	*P* value		
Self- management	1. I feel that I can cope well with the postpartum recovery process.	10.690	< 0.001	0.556	< 0.001	0.525	0.980
	2. I feel that I can cope well with taking care of my baby.	13.641	< 0.001	0.566	< 0.001	0.534	0.980
	3. I feel that I can schedule a postpartum follow-up visit to the health facility.	13.809	< 0.001	0.678	< 0.001	0.653	0.979
Social support	4. I feel that I can timely get access to help and support when I have my own health problems.	15.963	< 0.001	0.764	< 0.001	0.745	0.979
	5. I feel that I can timely get access to help and support when I have problems with taking care of my baby.	14.171	< 0.001	0.733	< 0.001	0.711	0.979
	6. I feel that my family cares for me.	11.942	< 0.001	0.725	< 0.001	0.707	0.979
	7. I feel that my family cares for my baby.	9.786	< 0.001	0.666	< 0.001	0.647	0.979
	8. I feel that the experiences of other mothers have provided a lot of help and support for me.	13.843	< 0.001	0.767	< 0.001	0.750	0.979
Facility- and community- based care	9. I feel that the medical staff in the hospital have maintained good service attitudes towards me.	15.452	< 0.001	0.841	< 0.001	0.830	0.978
	10. I feel that the postnatal care provided by the hospital can adequately meet my health needs.	15.661	< 0.001	0.789	< 0.001	0.771	0.978
	11. I feel that the medical staff in the hospital have sufficient time to answer my questions.	16.904	< 0.001	0.818	< 0.001	0.803	0.978
	12. I feel that the medical staff have carefully assessed my health status during hospitalization.	16.686	< 0.001	0.859	< 0.001	0.848	0.978
	13. I feel that the medical staff have carefully assessed my baby’s health status during hospitalization.	17.133	< 0.001	0.865	< 0.001	0.854	0.978
	14. I feel that the medical staff in the hospital have provided me with adequate information and guidance on postnatal physical and mental health care.	17.988	< 0.001	0.831	< 0.001	0.817	0.978
	15. I feel that the medical staff in the hospital have provided me with adequate information and guidance on newborn care.	18.654	< 0.001	0.870	< 0.001	0.859	0.978
	16. I feel that the medical staff in the hospital are very skilled.	15.809	< 0.001	0.847	< 0.001	0.836	0.978
	17. I feel that the postnatal health visitor has maintained good service attitudes towards me.	17.314	< 0.001	0.841	< 0.001	0.829	0.978
	18. I feel that the postnatal health visitor has carefully assessed my health status.	13.735	< 0.001	0.641	< 0.001	0.612	0.979
	19. I feel that the postnatal health visitor has carefully assessed my baby’s health status.	14.782	< 0.001	0.782	< 0.001	0.765	0.979
	20. I feel that the postnatal health visitor has provided me with adequate information and guidance on postnatal physical and mental health care.	12.593	< 0.001	0.738	< 0.001	0.717	0.979
	21. I feel that the postnatal health visitor has provided me with adequate information and guidance on newborn care.	16.929	< 0.001	0.781	< 0.001	0.763	0.979
	22. I feel that the postnatal health visitor is very skilled.	17.065	< 0.001	0.779	< 0.001	0.762	0.979
	23. I feel that the postnatal health visitor has sufficient time to answer my questions.	19.700	< 0.001	0.855	< 0.001	0.842	0.978
	24. I feel that the medical staff have carefully assessed my health status at the postnatal clinic.	19.667	< 0.001	0.906	< 0.001	0.898	0.978
	25. I feel that the medical staff have carefully assessed my baby’s health status at the postnatal clinic.	17.262	< 0.001	0.912	< 0.001	0.905	0.978
	26. I feel that the medical staff have provided adequate feedback or referral suggestion on my health examination results.	19.451	< 0.001	0.906	< 0.001	0.898	0.978
	27. I feel that the medical staff have provided adequate feedback or referral suggestion on my baby’s health examination results.	18.946	< 0.001	0.913	< 0.001	0.905	0.978
	28. I feel that the maternal and infant support facilities in the hospital can meet my needs for lactation and diaper change.	15.101	< 0.001	0.810	< 0.001	0.793	0.978
	29. I feel that the hospital has provided me with convenient services.	16.485	< 0.001	0.880	< 0.001	0.870	0.978
	30. I feel that the postnatal care provided by the community health center can fully meet my health needs.	17.644	< 0.001	0.864	< 0.001	0.852	0.978

**Table 3:** The KMO and Bartlett’s test results

Tests (n = 343)	Results	*P* value
KMO sample sufficiency	0.964	
Bartlett’s test of sphericity		
Chi-square value	11665.399	< 0.001
Degrees of freedom	435	

**Table 4:** Factor loadings of the items of the PCES

Item No	Item	Factor 1	Factor 2	Factor 3	Factor 4
1	I feel that I can cope well with the postpartum recovery process.	0.753			
2	I feel that I can cope well with taking care of my baby.	0.813			
18	I feel that the postnatal health visitor has carefully assessed my health status.		0.803		
19	I feel that the postnatal health visitor has carefully assessed my baby’s health status.		0.688		
3	I feel that I can schedule a postpartum follow-up visit to the health facility.			0.652	
6	I feel that my family cares for me.			0.821	
7	I feel that my family cares for my baby.			0.853	
8	I feel that the experiences of other mothers have provided a lot of help and support for me.			0.649	
9	I feel that the medical staff in the hospital have maintained good service attitudes towards me.			0.626	
4	I feel that I can timely get access to help and support when I have my own health problems.				0.602
5	I feel that I can timely get access to help and support when I have problems with taking care of my baby.				0.620
10	I feel that the postnatal care provided by the hospital can adequately meet my health needs.				0.547
11	I feel that the medical staff in the hospital have sufficient time to answer my questions.				0.634
12	I feel that the medical staff have carefully assessed my health status during hospitalization.				0.575
13	I feel that the medical staff have carefully assessed my baby’s health status during hospitalization.				0.574
14	I feel that the medical staff in the hospital have provided me with adequate information and guidance on postnatal physical and mental health care.				0.652
15	I feel that the medical staff in the hospital have provided me with adequate information and guidance on newborn care.				0.702
16	I feel that the medical staff in the hospital are very skilled.				0.550
17	I feel that the postnatal health visitor has maintained good service attitudes towards me.				0.530
20	I feel that the postnatal health visitor has provided me with adequate information and guidance on postnatal physical and mental health care.				0.536
21	I feel that the postnatal health visitor has provided me with adequate information and guidance on newborn care.				0.651
22	I feel that the postnatal health visitor is very skilled.				0.655
23	I feel that the postnatal health visitor has sufficient time to answer my questions.				0.770
24	I feel that the medical staff have carefully assessed my health status at the postnatal clinic.				0.792
25	I feel that the medical staff have carefully assessed my baby’s health status at the postnatal clinic.				0.754
26	I feel that the medical staff have provided adequate feedback or referral suggestion on my health examination results.				0.788
27	I feel that the medical staff have provided adequate feedback or referral suggestion on my baby’s health examination results.				0.746
28	I feel that the maternal and infant support facilities in the hospital can meet my needs for lactation and diaper change.				0.824
29	I feel that the hospital has provided me with convenient services.				0.777
30	I feel that the postnatal care provided by the community health center can fully meet my health needs.				0.757
Eigenvalue		2.492	3.845	5.780	10.622
Explained variance (%)		8.307	12.816	19.267	35.407
Total explained variance (%)		75.797			

**Table 5:** Internal consistency of the PCES and its correlation coefficient with the global item construct

Subscales	Number of items	Cronbach’s alpha	Split-half reliability	*r (95% CI)*
Subscale 1: Self-management	3	0.709	0.624	0.632 (0.526, 0.719)
Subscale 2: Social support	5	0.881	0.860	0.766 (0.684, 0.830)
Subscale 3: Facility- and community-based care	22	0.980	0.952	0.915 (0.883, 0.939)
Total	30	0.979	0.941	0.909 (0.874, 0.936)

## Discussion

In this study, we developed and validated a unique instrument (PCES) for measuring postpartum women’s overall experience of postnatal care through a rigorous process of item generation, scale development and scale evaluation. We considered this scale as having good content validity as the S-CVI/UA meet the pre-specified criteria. The 30-item PCES were determined through four item screening methods. The scale demonstrated excellent internal consistency reliability when tested in a Chinese population. Cronbach’s alpha for the overall PCES of 0.979 was above the preferred criterion of alpha *>* 0.70 for new instruments [48].

In our study, the results of the KMO index indicated adequate sample size and the factorability of the dataset. The EFA demonstrated a four-factor solution that was able to explain 75.797% of the total variance. Considering the practical significance, the four factors were grouped into three subscales including self-management, social support, and facility- and community-based care in the most meaningful way. The Pearson correlation coefficient between the total PCES score and the global item construct was 0.909, indicating that the scale had good convergent validity. Also, our study findings showed that the AVE of constructs exceeded 0.5 and construct reliability was greater than AVE, thus fulfilling the requirements of convergent validity. For discriminant validity, it was confirmed that the square root of the AVE scores were higher than the correlations values across constructs. Moreover, the CFA unfolded the suitability of the 3-factor model and the appropriate fit of its structural model for the study samples. Therefore, the final PCES instrument consisted of 30 items, with 3 items indicating women’s postpartum experience of self-management, 5 items representing experience of social support, and 22 items describing experience of facility- and community-based care.

Measurement of women’s experiences of maternity care is a critical component of care quality evaluation [28]. It is well-documented that the area of maternity care that is most in need of improvement is postnatal care [14, 50, 51]. Also, in a broader context, evaluation of consumer experiences of healthcare is important due to the positive association between consumer experiences and clinical outcomes [52]. In our study, the PCES was designed to be completed by women who had received facility- and community-based postnatal care, which is consistent with the increasing recognition of the value of women’s experiences and perceptions in evaluating quality of maternity care [28, 53, 54].

The instrument of PCES fills a scientific gap in the literature, given the dearth of overall experience of postnatal care measures for postpartum women. In contrast to most of the existing scales, the PCES can be used to assess women’s experience of postnatal care both during hospitalization and after discharge, which is in accordance with the WHO’s definition of the postnatal period, defined as the one beginning immediately after the birth of the baby and extending up to six weeks (42 days) [18]. The previous available tools for assessing women’s experience of postnatal care [23, 29, 55] were developed to evaluate maternal perceptions or experiences of early postnatal care during hospitalization; and these instruments did not cover postpartum women’s caring experiences after discharge. Moreover, the WOMen’s views of Birth Postnatal Satisfaction Questionnaire (WOMBPNSQ) was developed to assess women’s views of postnatal care [56], which however, had not been examined and applied in non-white populations and thus may not be suitable for Chinese cultures.

In our study, the three derived subscales of the PCES made conceptual sense, and echoed Faria-Schützer et al.’s findings [57]. Subscale 1 aimed to assess women’s coping experience of dealing with the routine of caring for her baby and for herself in the first few weeks after childbirth. This dimension was an important component of motherhood and could be challenged by many obstacles such as time, limited resources and difficulty to accept help, thus compromising women’s ability to care for themselves [58, 59]. Subscale 2 was designed to examine women’s experience of social support served by various interpersonal relationships. This dimension was a construct of the mother’s perception of the availability of others to provide emotional, psychological and material resources, and was also an important factor in enabling a successful transition to motherhood [60, 61]. The social support network for postpartum women means having family, friends and peers to turn to in times of need. Therefore, the items of subscale 2 involved essential resources provided by a social network to help women cope with their postnatal situations. Subscale 3 was intended to examine women’s experience of facility- and community-based care services in the postnatal period. These items measure components of experience of postnatal care identified by women as important in other studies [24, 62–65]. This dimension was addressed by focusing on the professional support provided by both health facility staffs and community health workers, including informational, emotional, technical and instrumental support. McLeish et al. also pointed out that health professionals play important roles in helping new mothers to cope, develop confidence, and to thrive [60]. Effective communication, respect and dignity, and emotional support constitute the key elements of experience of care [66]. In addition, provision of care, competent human resources, and essential physical resources available are also important factors influencing quality maternal and newborn care [66, 67], which may influence the way postnatal care is delivered and women’s evaluation of quality of care.

The PCES can be used as an outcome measure to evaluate quality of postnatal care, to compare and contrast quality of postnatal care across regions, populations, and care providers. Thus maternity care providers are enabled to identify specific aspects of postnatal care in need of quality improvement. The valid and reliable PCES may have implications for designing evidence-based intervention programs to improve women’s experiences of postnatal care. It also provides an opportunity for care providers and researchers to assess women’s experience of postnatal care before and after specific interventions.

The study has several limitations. First, we only included participants who underwent childbirth in one university hospital in urban China, which limits the generalisability of the results. As this scale was designed to be applicable to postnatal women in the general population, the items may not fully reflect the components of maternity care in specific situations such as care provided to high-risk women. Second, some selection bias was incurred as a result of using a convenience sample, in that women who lived in urban Shanghai and with a willingness to participate, may be over-represented and have perceived the experience of postnatal care more positively. Third, the self-administered scale was completed by women at 42 days postpartum and we did not include multiple postnatal time points. Therefore, we recommend that the performance of the PCES be tested at varying time points throughout the postnatal period so that women’s early health needs can be addressed in a timely manner. Fourth, as no other established scales were found to be the “gold standard” of scale measurement in this domain, we only compared the PCES measure to itself and one general item to establish its construct validity. Thus the validity evidence of the PCES was limited in this study and some additional validations, for instance, criterion validity and divergent validity, may be needed in future. Nonetheless, this study could be helpful in guiding further research, indicating that the validity and reliability of the PCES need to be further substantiated in diverse settings and within various cultural contexts.

## Conclusion

This study reports on the development and psychometric testing of the Chinese version of the Postnatal Care Experience Scale (PCES) to measure postpartum women’s experiences of care. This 30-item scale has indicated good construct validity and internal consistency reliability, which can be used as a tool to evaluate quality of postnatal care, to identify postpartum women with negative experiences, and for evaluating efforts to improve the quality of postnatal care. Future research should aim to use this scale in various populations to obtain further evidence for its validity and reliability.

## Data Availability

The datasets used and/or analyzed during the current study are available from the corresponding author on reasonable request.
